# Contrasting Ocular Manifestations of Xeroderma Pigmentosum in Siblings

**DOI:** 10.1002/ccr3.72553

**Published:** 2026-04-24

**Authors:** Mohammad Taher Rajabi, Ali Asadzadeh, Seyed Mohsen Rafizadeh, Amirhossein Aghajani, Amin Zand

**Affiliations:** ^1^ Department of Oculo‐Facial Plastic and Reconstructive Surgery, Farabi Eye Hospital Tehran University of Medical Sciences Tehran Iran; ^2^ School of Medicine Zanjan University of Medical Sciences Zanjan Iran

## Abstract

Xeroderma Pigmentosum (XP) can present with markedly different ocular severities even among siblings sharing the same genetic background. Early detection and preventive care are crucial to reduce the risk of vision‐ and life‐threatening malignancies.

## Case Presentation

**FIGURE 1:**
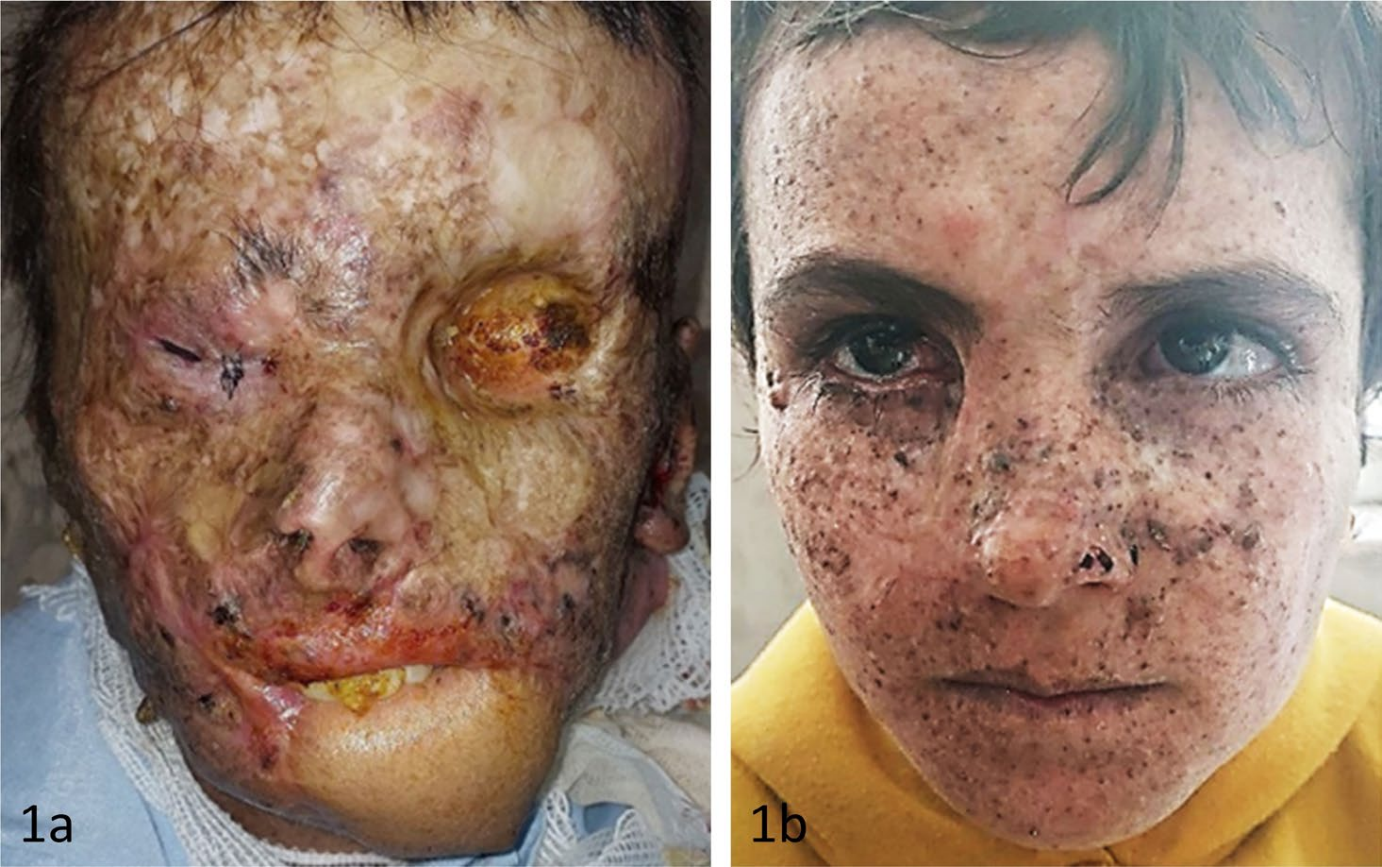
(a) The 14‐year‐old boy following left orbital exenteration and right‐eye tarsorrhaphy for exposure keratopathy. (b) The 10‐year‐old sister with mild dry eye disease and no neoplastic lesions.

Two siblings with XP exhibited strikingly different ocular phenotypes. The 14‐year‐old brother developed advanced disease with periocular squamous cell carcinoma (SCC) and orbital invasion, requiring left orbital exenteration. His course was further complicated by prior cutaneous SCC, exposure keratopathy, and keratitis necessitating tarsorrhaphy (Figure 1a). In contrast, his 10‐year‐old sister showed only mild ocular involvement, limited to dry eye disease with conjunctival xerosis and photophobia, managed conservatively with artificial tears. Both siblings receive prophylactic topical imiquimod and undergo regular ophthalmic follow‐up (Figure 1b).

## Discussion

XP is a rare autosomal recessive disorder characterized by nearly complete penetrance and caused by defects in the nucleotide excision repair (NER) pathway responsible for repairing ultraviolet (UV)–induced DNA damage. Impaired DNA repair leads to extreme UV sensitivity and a dramatically increased lifetime risk of malignancies, particularly in sun‐exposed tissues [1, 2]. Patients with XP are estimated to have a 1000‐fold higher risk of developing cutaneous cancers, contributing to significant morbidity and reduced life expectancy [1].

Beyond skin malignancies, individuals with XP exhibit a 10–20‐fold increased risk of systemic cancers, including breast, uterine, pancreatic, gastric, and mucosal carcinomas. Clinical manifestations vary widely and encompass mucocutaneous, neurological, and ophthalmic involvement. Common mucocutaneous features include photosensitivity, lentiginous pigmentation, actinic keratoses, skin atrophy, and seborrheic keratoses, with basal cell carcinoma (BCC), SCC, and melanoma representing the most frequent skin cancers. Neurological involvement occurs in approximately 20%–30% of patients and may include spasticity, hearing loss, seizures, and cognitive impairment [1].

Ocular involvement is reported in 40%–80% of XP cases and predominantly affects the anterior segment, which is highly susceptible to UV damage [1]. Frequent ophthalmic manifestations include photophobia, conjunctival erythema or pigmentation, pterygium, corneal opacities, cataracts, and eyelid malpositions. Prolonged UV exposure substantially increases the risk of ocular surface malignancies, including BCC, SCC, and conjunctival melanoma [3].

Prior studies highlight the heterogeneous clinical spectrum of XP. While benign cutaneous findings are common, malignant transformation occurs in a subset of patients [1]. Consistent with previous reports, both siblings in our study exhibited benign cutaneous manifestations; however, only one developed aggressive periocular SCC with orbital invasion. Ocular complications were similarly variable, with corneal involvement limited to the sibling with more severe disease. Neither sibling demonstrated neurological involvement, aligning with reported prevalence rates [1].

These findings underscore the marked phenotypic variability of XP, even among affected siblings. Management necessitates a multidisciplinary approach centered on strict sun avoidance, use of UV‐protective clothing and sunscreens, and timely intervention with preventive and therapeutic measures, including topical imiquimod, 5‐fluorouracil, lubricating agents such as methylcellulose, and antioxidant therapy, as well as early treatment of premalignant and malignant lesions [1, 2]. Although no curative therapy currently exists, early diagnosis, vigilant long‐term surveillance, and genetic counseling are critical for reducing disease‐related morbidity, preventing severe ocular complications, and improving overall survival.

## Author Contributions

**Mohammad Taher Rajabi:** conceptualization, investigation, supervision, validation, writing – review and editing. **Ali Asadzadeh:** data curation, writing – original draft. **Seyed Mohsen Rafizadeh:** writing – review and editing. **Amirhossein Aghajani:** writing – review and editing. **Amin Zand:** data curation, writing – original draft.

## Funding

The authors have nothing to report.

## Ethics Statement

The authors have nothing to report.

## Consent

The parent of these patients has given written informed consent for the publication of this manuscript and images.

## Conflicts of Interest

The authors declare no conflicts of interest.

## Data Availability

The data that support the findings of this study are available on request from the corresponding author. The data are not publicly available due to privacy or ethical restrictions.
